# Object-Level Visual-Text Correlation Graph Hashing for Unsupervised Cross-Modal Retrieval

**DOI:** 10.3390/s22082921

**Published:** 2022-04-11

**Authors:** Ge Shi, Feng Li, Lifang Wu, Yukun Chen

**Affiliations:** Faculty of Information Technology, Beijing University of Technology, Beijing 100124, China; shige@bjut.edu.cn (G.S.); lifeng9619@emails.bjut.edu.cn (F.L.); imbachenyukun@emails.bjut.edu.cn (Y.C.)

**Keywords:** cross-modal hash learning, deep model, hashing retrieval

## Abstract

The core of cross-modal hashing methods is to map high dimensional features into binary hash codes, which can then efficiently utilize the Hamming distance metric to enhance retrieval efficiency. Recent development emphasizes the advantages of the unsupervised cross-modal hashing technique, since it only relies on relevant information of the paired data, making it more applicable to real-world applications. However, two problems, that is intro-modality correlation and inter-modality correlation, still have not been fully considered. Intra-modality correlation describes the complex overall concept of a single modality and provides semantic relevance for retrieval tasks, while inter-modality correction refers to the relationship between different modalities. From our observation and hypothesis, the dependency relationship within the modality and between different modalities can be constructed at the object level, which can further improve cross-modal hashing retrieval accuracy. To this end, we propose a Visual-textful Correlation Graph Hashing (OVCGH) approach to mine the fine-grained object-level similarity in cross-modal data while suppressing noise interference. Specifically, a novel intra-modality correlation graph is designed to learn graph-level representations of different modalities, obtaining the dependency relationship of the image region to image region and the tag to tag in an unsupervised manner. Then, we design a visual-text dependency building module that can capture correlation semantic information between different modalities by modeling the dependency relationship between image object region and text tag. Extensive experiments on two widely used datasets verify the effectiveness of our proposed approach.

## 1. Introduction

The rapid development of multimedia data, such as text, image, video, and audio, has greatly enriched multimedia data diversity. Recently, cross-modal retrieval [1,2,3,4] has attracted more and more attention. It aims to retrieve instances of another modality given a query instance of one modal. However, the rapid growth of multimedia data puts forward higher requirements for retrieval efficiency. Many hashing methods have been proposed to tackle these challenges. Hashing methods can map high-dimensional features into binary hash codes, transforming information retrieval into Hamming distance calculation and greatly improving retrieval efficiency.

Due to the existence of “heterogeneous gaps” between different modalities, the similarity of different modalities cannot be measured directly. Existing hashing methods solve this problem mainly by mapping data of different modalities into a common Hamming space. The supervised cross-modal hashing methods [5,6,7] use a large amount of manually annotated data to capture the correlation information between different modalities so as to achieve satisfactory performance. However, the data annotation is labor intensive and high cost, making it infeasible in practical scenarios. Unlike the supervised method, the unsupervised hashing method only relies on weakly correlated information between modalities. It does not require annotated data, making it easier to deploy to real-world applications.

Most existing unsupervised cross-modal hashing methods utilize global-level intra-modality and inter-modality similarities to learn the hash functions. UGACH [8] made full use of GAN’s ability for unsupervised representation learning to exploit the underlying manifold structure of cross-modal data. UCH [9] combined generative adversarial networks and constructed two recurrent networks in an end-to-end framework to learn common feature representation and high-quality hash codes. DJSRH [10] improved the performance of retrieval by constructing the joint-semantic matrix and reconstructing with binary codes.

Driven by a large scale of image and text pairs with weakly semantic correlation, these methods have achieved great success. However, the text data consist of a series of tags provided by users, not annotated according to strict standards, and contains much noise. The direct use of the global level intra-modality and inter-modality similarity to learning hash functions will inevitably introduce a large amount of noise, leading to overfitting and degeneration models. In fact, both images and text have fine-grained granularities, i.e., objects in an image. Some object and tag pairs have slight similarities or even irrelevant at the fine-grained object level.

**Figure 1:**
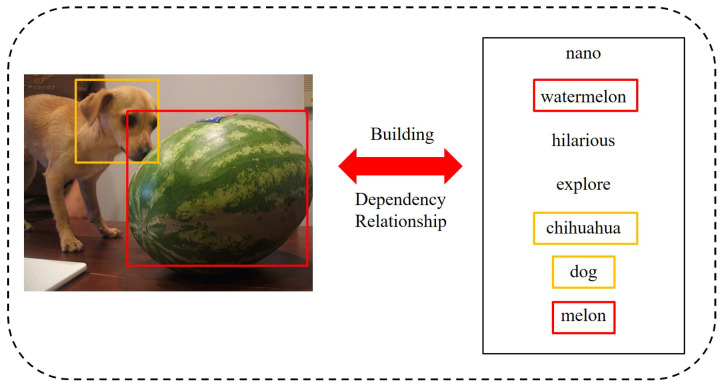
A pair of similar image text samples from the MIRFlickr dataset.

Figure 1 shows a set of image-text pairs in the MIRFlickr dataset. It can be seen that there is a corresponding relationship between the objects in the image and the tags in the text. Meanwhile, the unframed tags in the text are entirely unrelated to the image and are noise information. The existing methods based on global level mapping can not capture the similarities between objects and tags on fine-grained level, introducing much noise and resulting in non-optimal models.

To solve the above problems, we propose a novel Object-level Visual-text Correlation Graph Hashing (OVCGH) to model the corresponding relationship between image object regions and text tags. Specifically, based on the idea of deep graph infomax [11], OVCGH can construct an object-level visual correlation graph and a text correlation graph based on the image object region and the text tag, which not only learn the correlation between the objects of the single model data, but also do not ignore the overall information lost due to the object-level correlation. OVCGH constructs a fine-grained image object area and text tag relationship pair based on the existing correlation graph, which ensures that similar object-tag pairs in cross-modal data can have higher scores and effectively avoid the impact of noise on the model. Finally, the accurate hash codes are learned under the joint supervision of object-level dependency and global-level mapping. The contributions of this paper are summarized as follows:

- We propose a novel object-level visual-text correlation graph hashing (OVCGH) to mine the fine-grained object-level similarity existing in cross-modal data and suppress noise interference. The results on two public datasets show that our method achieves the best experimental performance.
- We design an object-level correlation graph building module that can construct the object-level similarity in the modal. It can construct the graph structure for the image modal and the text modal at the object-level in an unsupervised manner. Furthermore, the constructed graph structure contains the global information of its original semantic structure.
- We design a novel cross-modal dependency building module to build the object-level similarity between modalities while avoiding noise interference. It can model the dependency between the image object region and the text tag, ensuring that cross-modal data with similar objects can have a higher similarity score.

## 2. Related Work

The goal of cross-modal retrieval is to retrieve instances of another modality given a query instance of one modal. However, the amount of multimedia data is increasing rapidly, which sets higher requirements for improving the efficiency of retrieval. In order to solve this problem, many hashing methods [12,13,14] have been proposed to achieve fast and efficient cross-modal retrieval. Next, we divide the cross-modal hashing methods into two directions: unsupervised methods and supervised methods and introduce related work, respectively.

**Supervised cross-modal hashing methods** use labels to capture rich correlation information between different modalities to achieve cross-modal retrieval. Traditional supervised learning methods are mostly based on handcrafted features to learn semantic relevance in the common space. Bronstein et al. [14] proposed Cross-Modality Similarity Sensitive Hashing by using a boosted classification paradigm for hash learning. SCM [15] learned the hash function by constructing and maintaining the semantic similarity matrix. SePH [16] proposed a Semantics-Preserving Hashing method, which aimed to approximate the distribution of semantic labels and the distribution of hash codes on the Hamming space by minimizing the KL divergence.

Jiang et al. [17] proposed a Deep Cross-Modal Hashing method, which is one of the earliest methods to combine convolutional neural networks with cross-modal hashing. Li et al. [18] further improved this work and proposed Self-Supervised Adversarial Hashing, which was one of the first attempts to apply adversarial learning to the cross-modal hashing problem. Xu et al. [19] proposed a Graph Convolutional Hashing method, which used an association graph learning model to explore the inherent similarity structure among data. This method can help generate discriminants for the hash codes. Zhan et al. [7] proposed Supervised Hierarchical Deep Cross-modal Hashing, which implanted the similarity at each layer of the label hierarchy and the relatedness across different layers into the hash code learning. Although supervised methods achieved satisfactory performance, obtaining a large number of similar labels is usually expensive and very tricky, which makes supervised methods infeasible in real-world applications.

**Unsupervised cross-modal hashing methods** only rely on the relevant information in the paired data, making it easier to deploy to real-world applications. These methods usually learn hash codes by preserving inter-modality corrections. Traditional unsupervised cross-modal hashing methods mainly learn linear projection matrices by optimizing statistical analysis methods. Thompson et al. (CCA) [20] learned a subspace to maximize the correlation between two sets of heterogeneous data. IMH [21] proposed an Inter-Media Hashing method, which established a common Hamming space by maintaining the similarity between the inter-media and intra-media. Kumar et al. [22] extended Spectral Hash [23] to cross-modal retrieval and achieved better results.

Recently, deep learning techniques have also been developed to deal with cross-modal retrieval. UGACH [8] proposed an Unsupervised Generative Adversarial Cross-modal Hashing method, which made full use of GAN’s ability for unsupervised representation learning to exploit the underlying manifold structure of cross-modal data. As an improvement, UCH [9] combined generative adversarial networks and constructed two recurrent networks in an end-to-end framework to learn common feature representation and high-quality hash codes. Su et al. [10] proposed Deep Joint-Semantic Reconstruction Hashing, which further improved the performance of retrieval by constructing the joint-semantic matrix and reconstructing with binary codes.

Although unsupervised cross-modal hashing has obvious advantages in reducing the burden of data annotation, the accuracy is usually lower than a satisfactory level. Moreover, the existing methods mainly focus on mining the relationships between samples and consider them from the perspective of measuring the similarity of different samples without focusing on semantic information, so that the semantic similarity in the training data is not fully utilized, which motivates us to build the fine-grained dependence of the training data on the object level.

## 3. The Proposed Method

**Figure 2:**
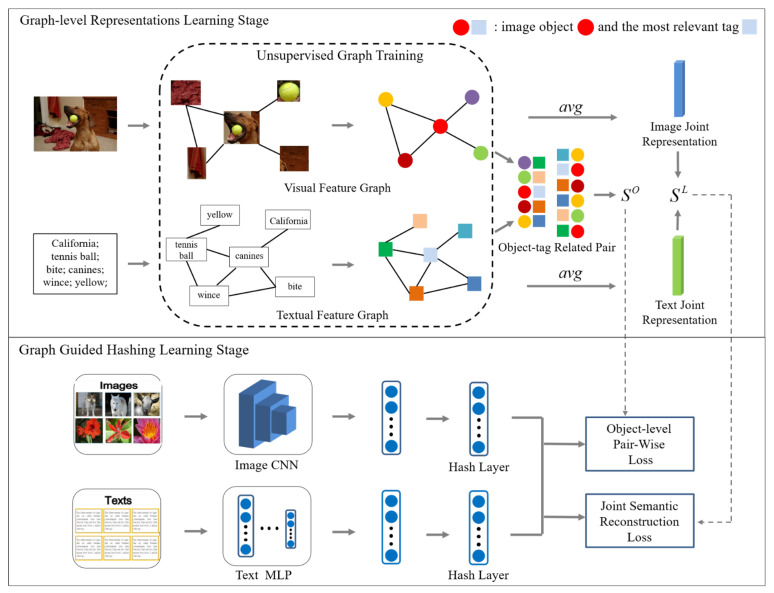
The overall framework of our proposed visual-textual correlation graph hashing (VCGH) approach.

The overall framework of our proposed approach is shown in Figure 2, which consists of two stages: (1) the graph-level representations learning stage; (2) the graph-guided hashing learning stage. Based on the similarity between image object regions and tags, the graph-level representations learning stage constructs two relation graph structures named visual feature graph and textual feature graph. The graph uses the strategy of maximizing the mutual information of local and global features to learn the graph-level representation of the image object regions and the text tags, respectively. In this training method, the nodes of the relation graph learn high-level representations that contain global information. Furthermore, the dependency between the object region and the tag can be modeled according to the existing relation graph structure. In this way, different modality data can be combined at a fine-grained level rather than as a whole. Through the feature interaction between the object-tag related pairs, the similarity score of the image and text at the object level $S^{O}$ can be obtained, which will be more conducive to judging the similarity between the image and the text. In addition, the joint representations that aggregate all nodes of the graph are used to calculate the global-level score $S^{L}$ between the image and the text. Finally, according to $S^{O}$ and $S^{L}$, the graph-guided hashing learning stage obtains the object-level pair-wise loss and joint semantic reconstruction loss, respectively, and the two loss functions are used to jointly optimize the learning of accurate hash codes.

### 3.1. Problem Definition

Next, we introduce some definitions used in this paper. For the cross-modal retrieval task, given the *p* instances of image-text pair input: $X={\left\{\left(x_{i}^{I},x_{i}^{T}\right)\right\}}_{i=1}^{p}$, where $x_{i}^{I}$ denotes the *i*th image sample and $x_{i}^{T}$ denotes the *i*th text sample. The goal of cross-modal hashing is to learn a hash function to generate hash codes of length *l* for different modalities, which maps data of different modalities into a common Hamming space for direct comparison.

In our method, a pre-trained visual feature extractor is used to encode the image object regions into feature vectors, and the visual feature extractor uses a pre-trained CNN network and an object detector (Faster-RCNN [24]). For each image $x_{i}^{I}$, the visual feature extractor encodes each image object region as a $d_{1}$ dimension visual feature vector $O_{i}={\left\{o_{i,j}\right\}}_{j=1}^{n_{i}}$, where $n_{i}$ is the number of object regions contained in the *i*th image, and $o_{i,j}$ is the *j*th visual feature vector of the image $x_{i}^{I}$. Each text $x_{i}^{T}$ is composed of multiple tags, so it is defined as $x_{i}^{T}={\left\{t_{i,j}\right\}}_{j=1}^{m_{i}}$, where $t_{i,j}$ is the *j*th tag and $m_{i}$ is the number of tags contained in the *i*th text.

### 3.2. Graph-Level Representations Learning

For each image and text, VCGH constructs visual undirected graph $G_{i}^{I}\;=\;\left(V_{i}^{I},E_{i}^{I}\right)$ and text undirected graph $G_{i}^{T}\;=\;\left(V_{i}^{T},E_{i}^{T}\right)$, respectively. For visual undirected graphs, $V^{I}$ is the set of all image object features related to $x_{i}^{I}$, and $E^{I}$ is the set of edges. The node set in $G^{I}$ is defined as follows:

(1) $$V_{i}^{I}={\left\{o_{i,j}\right\}}_{j=1}^{n_{i}}V^{I}\in R^{n_{i}\times d_{1}}$$

where $o_{i,j}$ defines each node in the node set, and two nodes are defined as neighbors if the cosine similarity between them is larger than zero. For $G^{T}$, each node defines the word embedding of the corresponding text tag, so the node set of the text undirected graph is defined as follows:

(2) $$V_{i}^{T}={\left\{F(t_{i,j})\right\}}_{j=1}^{m_{i}}V^{T}\in R^{m_{i}\times d_{2}}$$

where $v_{i,j}$ is a $d_{2}$ dimension word embedding representation of each text tag, which is mapped by the FastText model [25]. The FastText model is a widely used word-embedding extraction tool. Since the original text tags contain many uncommon words, the FastText model can be used to achieve better results. For each node in the text undirected graph, we use the same neighbor definition method as the visual undirected graph.

Leonardo et al. [26] pointed out that existing graph convolutional networks either use global nodes, which assume that each node is connected to other nodes, or use local nodes, which are connected to only a part of the nodes. The former ignores the structure of the graph, while the latter lacks global information. Inspired by [11], through the strategy of maximizing local and global mutual information, the relationship graph can capture not only the local structure information of the graph, but also model the overall knowledge of the graph. Therefore, Deep Graph Infomax [11] is used to learn the relationship between image object regions and text tags. Specifically, taking the image feature graph as an example, our training goal is to maximize the mutual information between each image object region and the entire image, so we can learn a mapping from the undirected visual graph to a high-level representation of each node:

(3) $$\varepsilon_{\theta_{I}}:\left(V_{i}^{I},E_{i}^{I}\right)\to Z_{i}^{I}$$

where $Z_{i}^{I}\;=\;\left\{z_{i,1},z_{i,2},\cdot \cdot \cdot ,z_{i,j},\cdot \cdot \cdot ,z_{i,n_{i}}\right\}$ is the high-level representation set of the nodes in the image feature graph, and the parameter $\theta_{I}$ is the learnable parameter of the image feature graph. Through this strategy, the image object region representation $o_{i,j}$ can be mapped to a *d* dimensional representation $z_{i,j}$ that contains the global information of the image. The text undirected graph can then be obtained by analogy as follows:

(4) $$\varepsilon_{\theta_{T}}:\left(V_{i}^{T},E_{i}^{T}\right)\to Z_{i}^{T}$$

where $Z_{i}^{I}\;=\;\left\{z_{i,1},z_{i,2},\cdot \cdot \cdot ,z_{i,j},\cdot \cdot \cdot ,z_{i,m_{i}}\right\}$ is the high-level representation set of the nodes in the textual feature graph, the parameter $\theta_{T}$ is the learnable parameter of the textual feature graph, and ${z^{\prime }}_{i,j}$ is a *d* dimension high-level representation containing the global information of the text.

When matching images and texts, the main judgment is based on the similarity between objects in the image and the text. Inspired by this, it is necessary to construct the similarity score between the image and the text at the object level. Thus, we construct a dependency between the image object region and the text tag based on the above graph structure. Specifically, based on the trained visual feature graph and textual feature graph, we can transform each image and each text into a d-dimensional vector set. Each vector in the set represents a high-level representation of the image object region or text tag, respectively. Furthermore, if an image object region is associated with a text tag, the object region and the tag will have a higher similarity score. The inner product method is used to measure the similarity score between the image region vector and the text tag vector. Therefore, the similarity score of the text-to-image is defined as follows:

(5) $$S=\frac{1}{m_{i}}\sum_{k=1}^{m_{i}}\max_{j\in [1,n_{i}]}z_{i,j}⊤{z^{\prime }}_{i,k}$$

where $\max_{j\in [1,n_{i}]}z_{i,j}^{T}{z^{\prime }}_{i,k}$ represents the similarity score between the *k*th tag in the text $x_{i}^{T}$ and the most relevant object region in the image $x_{i}^{I}$. Furthermore, the object-level similarity score of the text-to-image is obtained by the mean value method. Using the same method, we can obtain the similarity score of the image-to-text. The final object-level score is defined as follows:

(6) $$\begin{array}{c} S^{O}=\frac{1}{2}(\frac{1}{m_{i}}\sum_{k=1}^{m_{i}}\max_{j\in [1,n_{i}]}z_{i,j}⊤{z^{\prime }}_{i,k}\\ +\frac{1}{n_{i}}\sum_{k=1}^{n_{i}}\max_{j\in [1,m_{i}]}{z^{\prime }}_{i,j}⊤z_{i,k}) \end{array}$$

In addition to the object-level score, the global-level score is also considered to obtain the similarity between the image and the text. Specifically, after training the visual feature graph and the text feature graph, the global-level representation $r_{i}$ and ${r^{\prime }}_{i}$ of the image and text is calculated by the mean method:

(7) $$\begin{array}{cc} \; & r_{i}=\frac{1}{m_{i}}\sum_{k=1}^{m_{i}}z_{i,k}\\ \; & {r_{i}}^{\prime }=\frac{1}{n_{i}}\sum_{k=1}^{n_{i}}{z^{\prime }}_{i,k} \end{array}$$

Finally, we calculate the cosine similarity of $r_{i}$ and ${r^{\prime }}_{i}$ to obtain the global-level score $S^{G}=\cos\left(r_{i},{r^{\prime }}_{i}\right)$ between the image $x_{i}^{I}$ and the text $x_{i}^{T}$.

### 3.3. Graph Guided Hashing Learning

The goal of VCGH is to learn accurate hash codes for different modalities through the supervision of visual feature graphs and text feature graphs so that different modal data with similar semantic information are close in the public Hamming space. Specifically, the VCGH network contains two branch structures. Each branch contains an original space layer and a hash layer. The original space layer maps the original features of different modalities to the same common space for direct measurement, which can be expressed as:

(8) $$\phi_{c}\left(f\right)=\tanh\left(w_{c}f+b_{c}\right)$$

where $f=\{f^{I},f^{T}\}$ is the original feature of different modalities, i.e., $f^{I}$ for an image and $f^{T}$ for a text, $w_{c}$ denotes the weights, and $b_{c}$ is the bias parameter.

Through the hash layer, the common representations of different modalities are mapped to binary hash codes. which can be expressed as:

(9) $$h\left(f\right)=sigmoid(W_{h}\phi_{c}\left(f\right)+v))$$

where $W_{h}$ is the weight matrix and *v* is the bias parameter. Then a threshold function is used to get a final binary hash codes of length *l*:

(10) $$b\left(f\right)=sgn(h_{k}\left(f\right)-0.5),k=1,2,\cdots ,l$$

To preserve the original semantic information of the obtained hash codes as much as possible, VCGH uses the object-level score and the global-level score obtained from the graph as a joint guide to supervising network learning. Specifically, we first construct a similarity structure *H* by using the average inner product of different model hash codes:

(11) $$H=\frac{1}{l}b\left(f^{I}\right)⊤b\left(f^{T}\right)$$

where $b(f^{I})$ and $b(f^{T})$ are the hash codes of the image and text, respectively, and the average result is obtained by dividing the code length *l*. We minimize the $L_{2}$ loss of similar structure between the hash codes and the object level for different modal data, which is defined as the object-level pair-wise loss:

(12) $$L_{o}=\min{\left(H-S^{o}\right)}^{2}$$

For our proposed joint semantic reconstruction loss, we first calculate cosine similarity matrices $S^{I}=\cos\left(f^{I},f^{I}\right)$ and $S^{T}=\cos\left(f^{T},f^{T}\right)$ to represent the original semantic similarity structure of the image modality and text modality, respectively, and a weighted fusion strategy is used to jointly express the global-level similarity of different semantic information structures:

(13) $$S^{F}=\alpha S^{I}+\beta S^{T}+\gamma S^{G}$$

where α, β, and γ are weight parameters and $S^{F}$ is the global-level similarity matrix. Furthermore, we construct the joint semantic reconstruction loss to keep the hash codes discriminative from the intra-modal and inter-modal perspectives:

(14) $$\begin{array}{cc} L_{G}=\min{\left\|\lambda S^{F}-\cos\left(b\left(f^{I}\right),b\left(f^{T}\right)\right)\right\|}_{F}^{2} & \\ +\gamma \|\lambda S^{F}-\cos\left(b\left(f^{I}\right),b\left(f^{I}\right)\right){\|}_{F}^{2} & \\ +\eta \|\lambda S^{F}-\cos\left(b\left(f^{T}\right),b\left(f^{T}\right)\right){\|}_{F}^{2} & \end{array}$$

where ${\left\|\cdot \right\|}_{F}^{2}$ represents the Frobenius norm, λ is a hyper-parameter used to adjust the proportion of the global-level similarity structure, and λ and η are used to adjust the proportion of the intra-modal similarity. Finally, the loss function of VCGH is defined as follows:

(15) $$L=L_{o}+L_{G}$$

## 4. Experiments

In this section, we conduct some experiments on our proposed method. We first introduce the datasets, evaluation methods, and implementation details. Then, we compare and analyze the experimental results of VCGH with eight state-of-the-art methods. Finally, we conduct an ablation study to verify the importance of each part of VCGH.

### 4.1. Datasets and Compared Methods

The proposed VCGH and compared methods are evaluated on two widely used datasets: MIRFlickr [27] and NUS-WIDE [28].

**MIRFlickr** [27] contains 25,000 images and is annotated with 24 labels. Each image is associated with text tags and annotated with at least one label. Following [29], we use 20,015 image-text pairs in our experiments, where 2000 are preserved as the query set and the rest are used for retrieval database. A total of 5000 image-text pairs are randomly selected from the retrieval database as the training set.

**NUS-WIDE** [28] contains 269,648 images and is annotated with 81 labels. Each image is also associated with text tags and labeled with one or more of the labels. However, there are overlaps among the labels. Following [29], we selected the top 10 most frequent labels and the corresponding 186,577 image-text pairs as the database set. We randomly selected 2000 image-text pairs from the dataset as the query set and the others as the retrieval database, while 5000 image-text pairs are randomly selected from the retrieval database as the training set.

The proposed VCGH is compared with eight state-of-the-art methods. They are six unsupervised methods, i.e., CVH [22], PDH [30], CMFH [31], CCQ [32], DJSRH [10], MGAH [33], and two supervised methods, i.e., CMSSH [14] and SCM [15].

### 4.2. Evaluation Methods

In the experiment, we use two cross-modal retrieval tasks: using image queries to retrieve text in the database (image→text) and using text queries to retrieve images in the database (text→image). Specifically, we use the proposed VCGH method and all compared methods to obtain the hash codes of all query and retrieval database samples. We calculate the Hamming distance between the hash codes of the query and retrieval database samples and get a ranking list. Finally, we use two evaluation methods to evaluate the ranking list to verify the effectiveness of the retrieval: Mean Average Precision (MAP) and precision-recall curve (PRcurve). They are defined as follows:

MAP is a commonly used evaluation tool for information retrieval. It is computed as the mean of average precision (AP) of the retrieval results. The AP is computed as:

(16) $$AP=\frac{1}{R}\sum_{k=1}^{n}\frac{k}{R_{k}}\times rel_{k}$$

where *n* is the size of database, *R* is the number of relevant images or text in the database, $R_{k}$ is the number of related images or text in the ranking list of length *k*, and $rel_{k}$ means if the *k*th sample and the query sample have the same label then $rel_{k}\;=\;1$, otherwise $rel_{k}=0$.

The PR curve is obtained according to the precision and recall rate corresponding to each retrieval position.

### 4.3. Implementation Details

In this section, we present the implementation details of our VCGH in the experiments. For the graph-level representation learning stage, we use the pre-trained object detection model FasterRCNN [24] to detect the object regions in the image and use the 19-layer VGGNet [34] pre-trained on the ImageNet dataset [35] to get the 4096-dimensional features of the object region for each image. Then, the Deep Graph Infomax model is trained for 30 epochs to map the image object region into a 512-dimensional graph-level representation. For text, we use the FastText model [25] to extract the 300-dimensions text feature of each tag and then use the same training method to obtain a 512-dimensional text graph-level representation. For the graph-guided hashing learning stage, we use 19-layer VGGNet and Multilayer Perceptron as the backbone network of our image and text modalities, respectively, and then, a fully connected layer of length is used to obtain the hash codes. We train the network using the mini-batch method and set the batch size to 32. The learning rates are set to 0.001 for the image model and 0.01 for the text model. Moreover, the network is trained for 80 epochs and decreased by a factor of 10 at 60 epochs.

In addition, for a fair comparison, we apply the same implementation of compared methods provided by the authors and follow their best settings to perform the experiments. We use the same image and text features for all compared methods and use a 19-layer VGGNet pre-trained on the ImageNet dataset to extract image deep features. We finally apply the bag of words model to extract Bow text features for texts.

### 4.4. Experiment Results

Table 1 and Table 2 show the MAP scores from 16 to 128 hash bits on the MIRFlickr dataset and NUS-WIDE dataset. From the results, we can clearly observe that VCGH significantly outperforms the eight state-of-the-art methods on the two datasets. Specifically, on the MIRFlickr dataset, our method achieves the best average MAP score of 0.712 on the image→text task and 0.719 on the text→image task. Compared to the best unsupervised method MGAH, our method achieves a performance improvement from 0.696 to 0.712 on the image→text task and from 0.681 to 0.719 on the text→image task. On the NUS-WIDE dataset, our method also achieves the best average MAP score of 0.628 on the image→text task and 0.632 on the text→image task. These improvements demonstrate the effectiveness of modeling the dependency relationship between the image object region and the text tag by constructing visual feature graphs and text feature graphs.

**Figure 3:**
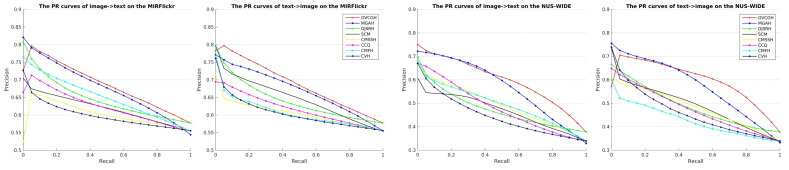
The PR curves on the MIRFlickr and NUS-WIDE datasets with 16-bit hash codes.

Figure 3 shows the precision-recall curves of 16-bit hash codes on two datasets. We can observe that our proposed VCGH approach also maintains the best accuracy on image→text tasks and text→image tasks among all compared methods, which further demonstrates the effectiveness of our proposed method.

### 4.5. Ablation Study

In this section, we compare three baseline methods to verify the importance of each part in VCGH.

*Baseline*: we design a baseline method without using visual feature graphs and textual feature graphs named Baseline. Specifically, Baseline trains the network using the joint semantic reconstruction loss without containing the global-level score of the relational graph, $S=\alpha S^{I}+\beta S^{T}$, so the training objective function is:

(17) $$\begin{array}{cc} L=\min\|\lambda S-\cos\left(b\left(f^{I}\right),b\left(f^{T}\right)\right){\|}_{F}^{2} & \\ +\gamma \|\lambda S-\cos\left(b\left(f^{I}\right),b\left(f^{I}\right)\right){\|}_{F}^{2} & \\ +\eta \|\lambda S-\cos\left(b\left(f^{T}\right),b\left(f^{T}\right)\right){\|}_{F}^{2} & \end{array}$$

*Baseline-global*: We introduce the global-level score obtained from the relationship graph on the basis of Baseline, so the objective training function of Baseline-global is defined in Formula (14).

*Baseline-object*: We add an object-level paired-wise loss based on the Baseline, which is defined in Equation (12).

Table 3 shows the MAP scores by using different variants of the proposed VCGH on MIRFlickr and NUS-WIDE. We can clearly observe that Baseline-global achieves an average performance improvement of 0.014 and 0.01 compared with Baseline for the image→text task and text→image task on the MIRFlickr dataset, which demonstrates the effectiveness of the proposed visual feature graph and text feature graph. We can also see an average performance improvement of 0.048 and 0.029 for the two tasks on the NUS-WIDE dataset, which further demonstrates the above conclusion. By comparing Baseline-object with Baseline, we can see that the average performance of the two tasks on the MIRFlickr dataset increased by 0.016 and 0.017, and the NUS-WIDE dataset also increased by 0.01 and 0.018, which further demonstrates the effectiveness of building the dependency relationship between the image object region and the text tag. By comparing our proposed VCGH method with Baseline-object, We can see that the average performance of the two tasks on the MIRFlickr dataset increased by 0.015 and 0.016, and the NUS-WIDE dataset also increased by 0.016 and 0.016, which demonstrates that the object-level score and the global-level score complement each other and further improve the accuracy of cross-modal retrieval.

## 5. Conclusions

This paper proposes a novel visual-textful correlation graph hashing (VCGH) approach for unsupervised cross-modal retrieval. VCGH uses an unsupervised method to construct visual feature graphs and text feature graphs for different modalities. The proposed features graphs model the information association in the single modality to obtain more semantic information graph-level representations. Furthermore, based on the existing graph structure, VCGH can model the dependency relationship between the image object region and the text tag and improves the accuracy of cross-modal retrieval from the perspective of global-level scores and object-level scores. Experimental results on two widely used datasets demonstrate the superiority of the proposed method, which illustrates the positive effect of mining semantic information to explore cross-modal retrieval tasks.

With the widespread popularity and application of multi-modal data, in the future, we will further extend our approach to more modals for cross-modal hash retrieval, and we wonder whether the semantic information would have great impact on multi-modal scenarios performances.

## Figures and Tables

**Table 1:** The MAP scores of two retrieval tasks on the MIRFlickr dataset with different lengths of hash codes. The best results are highlighted in bold.

Method	Image→Text				Text→Image			
	16	32	64	128	16	32	64	128
CVH [22]	0.602	0.587	0.578	0.572	0.607	0.591	0.581	0.574
PDH [30]	0.623	0.624	0.621	0.626	0.627	0.628	0.628	0.629
CMFH [31]	0.659	0.660	0.663	0.653	0.611	0.606	0.575	0.563
CCQ [32]	0.637	0.639	0.639	0.638	0.628	0.628	0.622	0.618
CMSSH [14]	0.611	0.602	0.599	0.591	0.612	0.604	0.592	0.585
SCM [15]	0.636	0.64	0.641	0.643	0.661	0.664	0.668	0.670
DJSRH [10]	0.659	0.661	0.675	0.684	0.655	0.671	0.673	0.685
MGAH [33]	0.685	0.693	0.704	0.702	0.673	0.676	0.686	0.690
VCGH	**0.701**	**0.711**	**0.714**	**0.723**	**0.705**	**0.713**	**0.725**	**0.733**

**Table 2:** The MAP scores of two retrieval tasks on the NUS-WIDE dataset with different lengths of hash codes. The best results are highlighted in bold.

Method	Image→Text				Text→Image			
	16	32	64	128	16	32	64	128
CVH [22]	0.458	0.432	0.410	0.392	0.474	0.445	0.419	0.398
PDH [30]	0.475	0.484	0.480	0.490	0.489	0.512	0.507	0.517
CMFH [31]	0.517	0.550	0.547	0.520	0.439	0.416	0.377	0.349
CCQ [32]	0.504	0.505	0.506	0.505	0.499	0.496	0.492	0.488
CMSSH [14]	0.512	0.470	0.479	0.466	0.519	0.498	0.456	0.488
SCM [15]	0.517	0.514	0.518	0.518	0.518	0.510	0.517	0.518
DJSRH [10]	0.503	0.517	0.528	0.554	0.526	0.541	0.539	0.570
MGAH [33]	0.613	0.623	0.628	0.631	0.603	0.614	0.640	0.641
VCGH	**0.615**	**0.628**	**0.631**	**0.639**	**0.617**	**0.628**	**0.635**	**0.648**

**Table 3:** The MAP scores by using different variants of the proposed VCGH on MIRFlickr and NUS-WIDE. The best results are highlighted in bold.

Task	Method	MIRFlickr				NUS-WIDE			
		16	32	64	128	16	32	64	128
image→text	Baseline	0.656	0.682	0.689	0.698	0.507	0.559	0.569	0.581
	Baseline-global	0.681	0.688	0.692	0.703	0.589	0.593	0.611	0.615
	Baseline-object	0.686	0.691	0.699	0.710	0.594	0.611	0.619	0.623
	VCGH	**0.701**	**0.711**	**0.714**	**0.723**	**0.615**	**0.628**	**0.631**	**0.639**
text→image	Baseline	0.664	0.690	0.691	0.700	0.498	0.583	0.576	0.619
	Baseline-global	0.680	0.683	0.687	0.694	0.581	0.588	0.610	0.614
	Baseline-object	0.691	0.696	0.710	0.718	0.597	0.616	0.621	0.628
	VCGH	**0.705**	**0.713**	**0.725**	**0.733**	**0.617**	**0.628**	**0.635**	**0.648**

## Data Availability

Data sharing is not applicable to this paper as no new data were created or analyzed in this study.
